# MRI-based measurement of inner ear fluids reveals increased endolymph volume variability in patients with endolymphatic hydrops and hearing instability

**DOI:** 10.1038/s41598-025-06083-w

**Published:** 2025-07-02

**Authors:** Julia Telischi, Dillon Strepay, Bing Li, Jennifer Chisholm, Hui Cheng, Carmen Brewer, Li-Yueh Hsu, John Butman, Michael Hoa

**Affiliations:** 1 Auditory Development and Restoration Program, Neurotology Branch, National Institute on Deafness and Other Communication Disorders (NIDCD), National Institutes of Health, Bethesda, MD USA; 2 Radiology and Imaging Sciences, NIH Clinical Center, National Institutes of Health, Bethesda, MD USA; 3 Auditory and Vestibular Clinical Research Section (AVCRS), National Institute on Deafness and Other Communication Disorders, NIH, Bethesda, MD USA; 4 Data Science Core, National Institute on Deafness and Other Communication Disorders, NIH, Bethesda, MD 20892 USA; 5 Porter Neuroscience Research Center, 35 Convent Dr., Rm 1F226, Bethesda, MD 20892-3745 USA

**Keywords:** Biomarkers, Translational research, Medical imaging

## Abstract

Lack of proven biomarkers for hearing instability (HI) disorders leads to difficulty and delays in diagnosis. Given the association of HI with endolymphatic hydrops (EH), imaging-based techniques for quantification of endolymph and perilymph may be useful for improved clinical management. This study longitudinally characterizes variations in endolymph fluid volume and correlations with hearing changes. This prospective cohort study (2021–2024) followed 14 patients (age 29–73) meeting criteria for HI over 15 months and 12 healthy volunteers (age 22–75). Contrast Enhanced Delayed (CED)-MRI using 3D FLAIR and STIR sequences was performed every 3–6 months, under a deep phenotyping protocol including standard hearing threshold testing. Perilymph and endolymph volumes were quantified using a semi-automatic MRI processing and analysis pipeline. Individual endolymph-to-perilymph (E/P) ratio variance over time was higher in ears with fluctuating hearing and MRI-designated EH (Mann-Whitney U Test: *U* = 39, 27; *p* = 0.01, 0.003 respectively). On linear regression, increasing vestibule E/P ratio correlated with worse hearing (*Estimate* = 45.31, *SE =* 7.88, *p* < 0.001) and visit-to-visit change in E/P ratio increased with shifts to worse hearing thresholds (*Estimate* = 141.84, *SE* = 45.69, *p* = 0.003). Longitudinal assessment of HI patient imaging therefore allows detection of quantifiable fluctuations in EH that correlate with hearing changes. This methodology could improve monitoring of HI and evaluation of potential therapies. Trial registration number: NCT04806282.

**Supplementary Information:**

The online version contains supplementary material available at 10.1038/s41598-025-06083-w.

## Introduction

Hearing instability (HI) disorders encompass a range of diseases that are characterized by fluctuating or sudden hearing loss. These include Meniere’s disease (MD), sudden sensorineural hearing loss (SSNHL), and autoimmune inner ear disease (AIED), with MD being the most prevalent^1–5^. Diagnosing and treating HI is challenging due to inconsistency and heterogeneity between and within patients and the lack of adequate biomarkers. While treatments are available for associated vestibular symptoms, effective therapies for hearing loss in these disorders remain limited^2^.

**Fig. 1:**
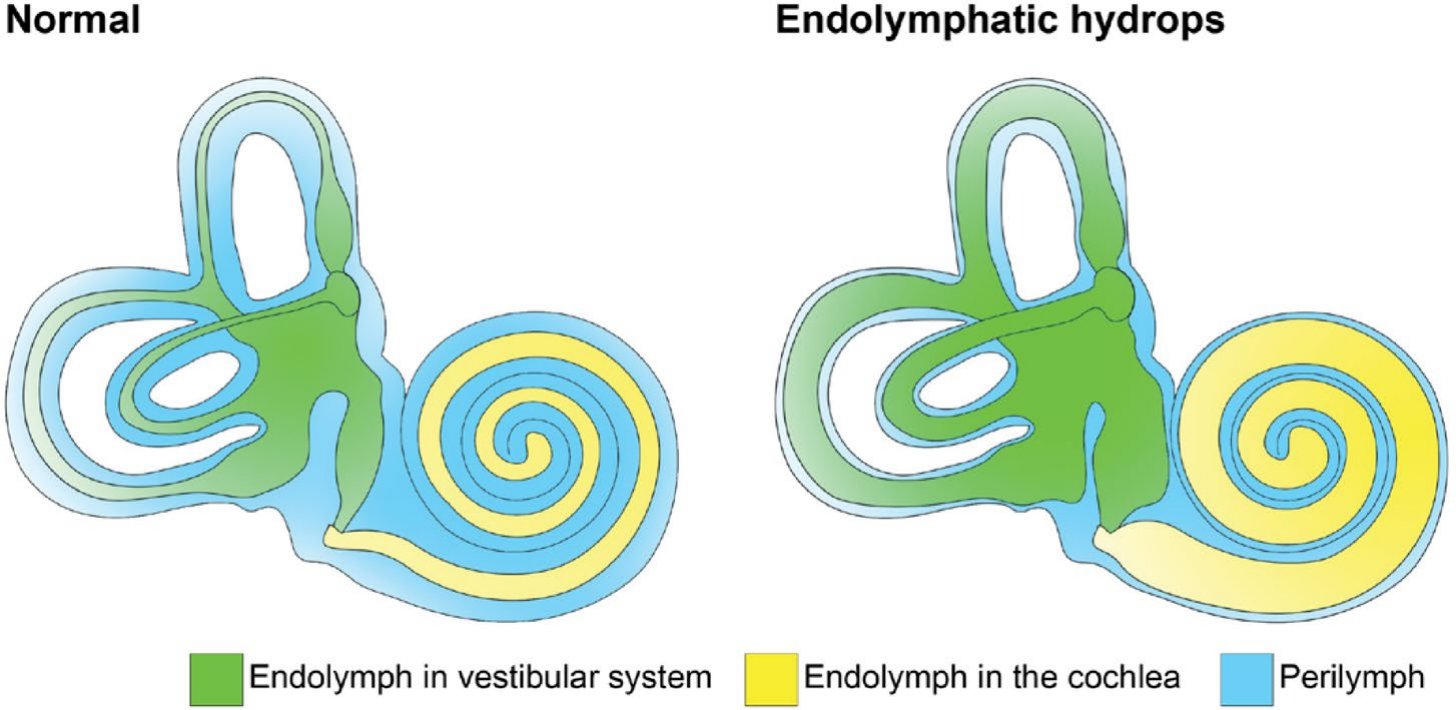
Graphic representation of endolymphatic hydrops in the inner ear. Perilymph is shown in light blue. Endolymph in the vestibular system is shown in green and is yellow in the cochlea. The endolymph comprises a significantly larger proportion of the inner ear space in the hydropic structure on the right.

Endolymphatic hydrops (EH), an expansion of the endolymphatic fluid space within the inner ear (Fig. 1), has been associated with conditions of HI^6–10^. While the mechanism of this phenomenon remains unknown, prevailing theories include abnormal flow or production of endolymph and disruption of cochlear ionic homeostasis^11^. EH was first discovered in human temporal bone sections of Meniere’s patients in 1938^12,13^. Since then, techniques have been developed to detect EH in vivo using T2-weighted or short tau inversion recovery (STIR) and contrast-enhanced delayed fluid attenuated inversion recovery (CED-FLAIR) MRI^14–17^. Gadolinium based contrast agents selectively accumulate in the perilymph, and do not cross into the endolymph due to their large molecular weight preventing diffusion across tight junctions^18–20^. Therefore, CED-FLAIR sequences show hyperintense signal in the perilymph only, compared to total labyrinthine fluid detected on STIR sequences.

Several grading schemes for EH have been proposed, including the Baráth classification and the Nakashima classification, but their reliability and application is inconsistent^21–25^. As a consequence, more objective and consistent tools are needed. MRI-based fluid volume quantification is a more precise measure for assessing the extent of EH shown to be reliable and accurate and previously used to compare endolymph volume to hearing level^15,25–27^. However, most previous studies use a single timepoint and include only patients with MD^15,25–27^. Given the heterogeneous and fluctuating nature of the disease course, we hypothesize that endolymph volume is more variable in ears with unstable hearing and that demonstrate EH. However, longer protocols are necessary to better characterize the role of EH in hearing instability. This work uniquely observes this population longitudinally to investigate the relationship between volumetric changes in EH and hearing outcomes, and to explore the potential of endolymph volume as a diagnostic and therapeutic biomarker for HI.

## Results

### Cohort demographics

Fourteen affected patients (28 ears) with 3–7 visits and 12 healthy volunteers (HVs; 24 ears) with 1–2 visits were included (142 affected instances and 38 HV instances). Demographics (Table 1) showed equal gender distribution. Mean ages were 48±13 years for affected patients and 50±21 years for HVs.

**Table 1:** HI and HV cohort demographics.

		Affected Patients	Healthy Volunteers
Sex	Female	50.0% (*N* = 7)	50.0% (*N* = 6)
	Male	50.0% (*N* = 7)	50.0% (*N* = 6)
Race	Asian	7.1% (*N* = 1)	25.0% (*N* = 3)
	Black or African American	7.1% (*N* = 1)	16.7% (*N* = 2)
	White	85.7% (*N* = 12)	58.3% (*N* = 7)
Age	Mean (SD)	48 (13)	50 (21)
Diagnoses by patient	AIED	7.14% (*N* = 1)	-
	HI - NOS	7.14% (*N* = 1)	-
	MD	57.14% (*N* = 8)	-
	SSNHL	7.14% (*N* = 1)	-
	Multiple Diagnoses	21.43% (*N* = 3)	-
	Comorbid Migraine	14.29% (*N* = 2)	-
Diagnoses by ear	Meniere’s Disease	50.00% (*N* = 14)	-
	SSNHL	14.29% (*N* = 4)	-
	AIED	14.29% (*N* = 4)	-
	HI-NOS	3.57% (*N* = 1)	-
	Unaffected	28.57% (*N* = 8)	-
	*Ears with > 1 diagnosis	10.71% (*N* = 3)	-
Laterality	Bilateral disease	42.86% (*N* = 6)	-
	Unilateral disease	57.14% (*N* = 8)	-
	Affected Right Ears	71.43% (*N* = 10)	-
	Affected Left Ears	71.43% (*N* = 10)	-

Distributions by sex, race, ethnicity, and age (total *N* = 14 HI affected patients; *N* = 12 HV patients). No patients identified as Latino or Hispanic ethnicity. Description of diagnoses by patient (total *N* = 14 affected patients) and by ear (total *N* = 28 ears). Patients with a diagnosis of “multiple diagnoses” had > 1 diagnosis or May have different diagnoses for left and right ear. Comorbid migraine was considered separately to the main diagnosis. Values in the diagnosis by ear section do not sum to 100% as several ears had more than one diagnosis associated. AIED = autoimmune inner ear disease; HI-NOS = hearing instability-not otherwise specified; md = meniere’s disease; ssnhl = sudden sensorineural hearing loss. *Counted separately from the other rows in this section.

Diagnoses included MD (*N* = 8 patients), idiopathic SSNHL (*N* = 1), AIED (*N* = 1), and hearing instability not otherwise specified (HI-NOS; *N* = 1). Three patients had ‘mixed’ diagnoses (> 1 diagnosis per ear or different diagnoses for left and right ear). Two patients had comorbid migraine. Unaffected ears from patients with unilateral disease were also included.

### Hearing instability coincides with MRI-determined EH

Hydrops was present on CED-MRI for 46 (32.4%) instances and absent for 96 (67.6%). Hearing was stable for 92 (64.8%) and unstable in 22 (15.5%; 11 improved, 11 worsened) instances; 28 (19.7%) baseline instances lacked a stability designation. Unstable hearing was significantly more likely to coincide with MRI-determined EH (*N* = 15; 39.5%) than without EH (*N* = 7; 9.2%; Fisher’s Exact Test: odds ratio = 6.43, 95% CI [2.3,17.7], *p* < 0.001). Abnormalities in pre- and immediately post-contrast imaging did not correspond to increased likelihood of hearing instability or EH (Supplemental Analysis 1).

### Semi-automated fluid volume quantification reliability

Total volume (TV) showed minimal variance across visits. TV and endolymph to perilymph (E/P) ratio measurements correlated well between two observers (cochlea E/P ratio intraclass correlation coefficient (ICC) = 0.90, vestibule E/P ratio ICC = 0.88; cochlea TV ICC = 0.86, vestibule TV ICC = 0.82). Expanded description of reliability analysis is reported in Supplemental Analysis 2 and Supplemental Fig. 4.

Neither gadolinium dose nor delay time affected perilymph volume measurement (Supplemental Fig. 5).

### Endolymph and Perilymph volumes vary more in unstable and hydropic ears

**Fig. 2:**
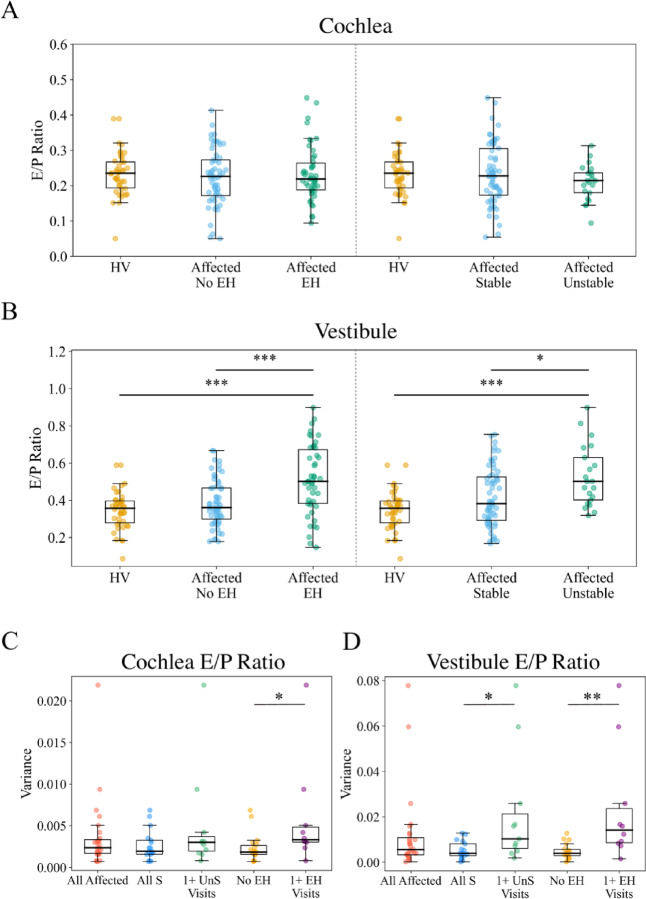
E/P Ratio Comparisons. E/P ratio for all instances is shown in (**A**) and (**B**). (**A**) Affected instances are grouped by hydrops status and compared to HVs left of the dotted lines and by hearing stability status on the right for cochlear E/P ratio. (**B**) Affected instances are grouped by hydrops status and compared to HVs left of the dotted lines and by hearing stability status on the right for vestibular E/P ratio. Significant differences in values tested by Kruskall-Wallis H Test and post-hoc Mann-Whitney U Test are represented by solid black lines. (**C-D**) Variances in inner ear fluid volumes for individual ears over the course of their participation in the study protocol. (**C**) Cochlea E/P ratio variances were significantly different based on the presence of EH, but not hearing stability (Mann-Whitney U Test: *p* = 0.01, 0.30 respectively). (**D**) E/P ratio variances were significantly different based on both the presence of EH and hearing stability in the vestibule (Mann-Whitney U Test: *p* = 0.003, 0.01 respectively). ***p < 0.001, **p < 0.01, *p < 0.05; EH = endolymphatic hydrops; S = stable; UnS = unstable; 1 + = at least 1 episode.

E/P ratio values were compared between HVs and affected ears grouped by hydrops status and by hearing stability. Inner ear fluid volumes are detailed in Supplemental Table 2 in comparison to previous literature, and statistical testing details are presented in Supplemental Table 3. Mean cochlear TV and E/P ratio were 93.18 ± 10.47 µL and 0.24 ± 0.05 in HVs, and 91.42 ± 11.86 µL and 0.23 ± 0.07 in affected ears, respectively. Cochlear E/P ratio distribution did not differ based on hydrops status (Fig. 2A). Mean vestibular TV was 59.57 ± 8.67 µL for HV instances and 57.39 ± 9.32 µL for affected instances, while vestibular E/P ratios were 0.35 ± 0.086 and 0.43 ± 0.15, respectively. Vestibular E/P ratios were significantly higher in affected instances with EH than HVs and non-EH affected instances (Fig. 2B; *p* < 0.001), confirming a higher proportion of endolymph in hydropic ears. Grouping by hearing stability (Supplemental Table 4) showed cochlear E/P ratios did not differ significantly between stable or unstable ears and HVs (Fig. 2A; *p* = 0.23), but vestibular E/P ratios were elevated in unstable instances compared to HVs and stable instances (Fig. 2B; *p* < 0.001 and *p* = 0.025 respectively). Overall, E/P ratios in the cochlea did not distinguish well between different patient groupings, however vestibular E/P ratios demonstrated differences based on hydrops status and hearing stability.

Fluid volume variance across the study protocol was analyzed to identify longitudinal patterns of volume shifts. Ears with at least one visit of MRI-determined EH showed significantly higher cochlear and vestibular E/P ratio variance than ears without EH (Fig. 2C-D; *p* = 0.01, 0.003). E/P ratio varied significantly more in ears with unstable hearing than ears with stable hearing in the vestibule (Fig. 2D; *p* = 0.01) but not the cochlea (Fig. 2C; *p* = 0.30). In ears with at least one visit of MRI-determined EH across the study protocol, cochlear and vestibular E/P ratios varied significantly more (Fig. 2C-D; *p* = 0.01, 0.003). These data support the hypothesis that increased E/P ratio variance, particularly in the vestibule, is associated with hearing instability and demonstration of EH.

### Hearing correlates with fluid volumes

**Fig. 3:**
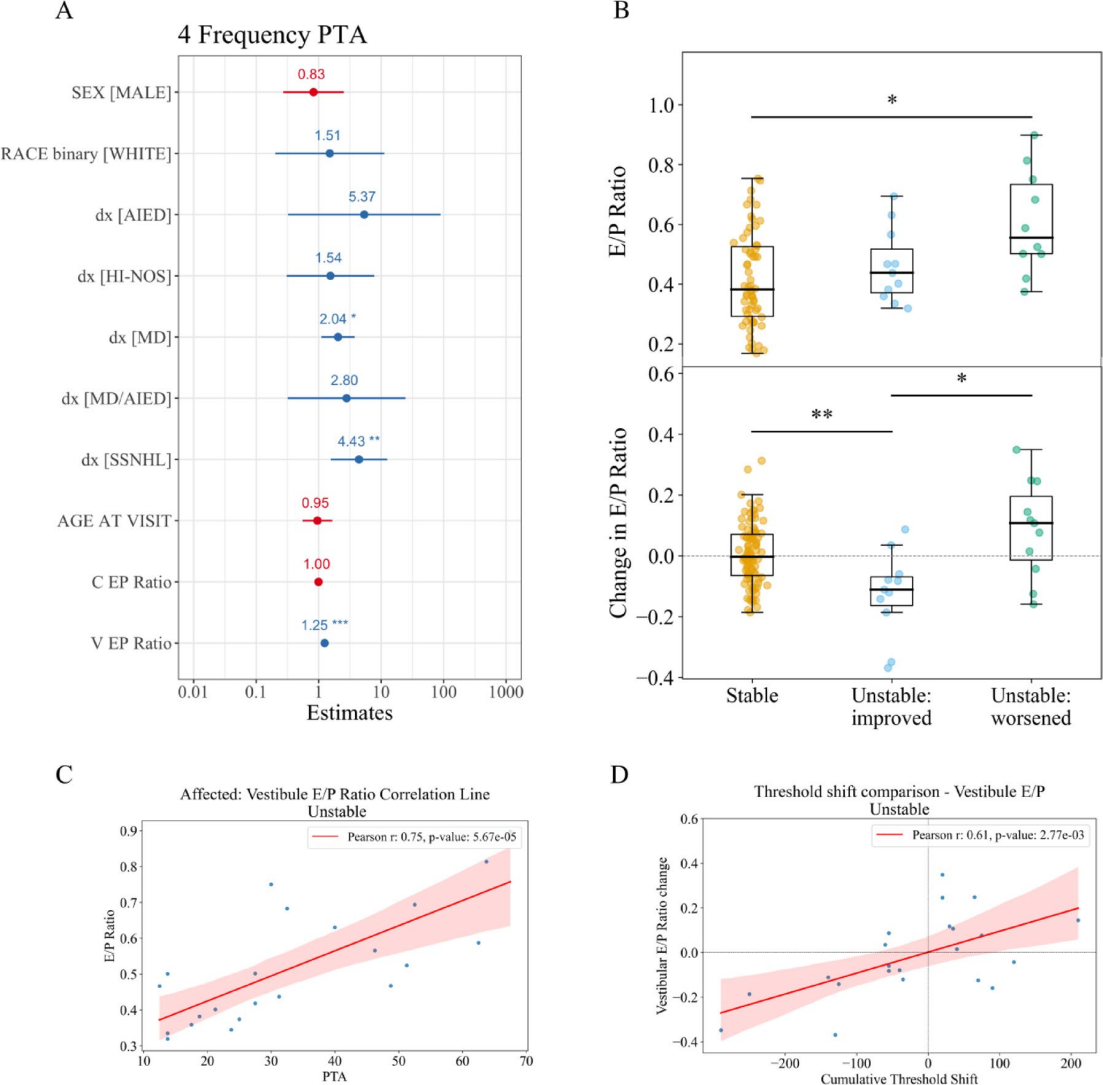
Correlations and regression modeling show the relationship between volume parameters and hearing level. (**A**) Coefficient plot of regression coefficients of all fixed effects included in a mixed linear regression model predicting pure tone average (PTA). (**B**) E/P Ratio and visit-to-visit change grouped by hearing stability status and unstable visits were further stratified by overall improvement or worsening in hearing. E/P ratio was significantly higher in unstable-worsened instances (Kruskal-Wallis *H* statistic = 9.51, *p* = 0.0086; Mann-Whitney *U* statistic = 131, adjusted *p* = 0.011). Compared to stable ears, visit-to-visit E/P ratio change was more negative in ears with improved hearing (Kruskal–Wallis *H* statistic = 12.41, *p* = 0.0020; adjusted Mann–Whitney *U* statistic = 787, *p* = 0.0082). Compared to ears with improved hearing, visit-to-visit E/P ratio change was more positive in ears with worsened hearing and (adjusted Mann–Whitney *U* statistic = 20, *p* = 0.026). (**C**) Correlation between vestibular E/P ratio and pure tone average (PTA) for instances with unstable hearing (Pearson’s correlation: *r* = 0.75, *p* < 0.001). (**D**) Correlation between vestibular E/P ratio visit-to-visit change and cumulative threshold shift for ears with unstable hearing (Pearson’s correlation: *r* = 0.61, *p* = 0.003). Visit-to-visit threshold shift was calculated as the sum of hearing threshold differences larger than 10dB HL for each frequency compared to the most recent prior audiogram. C = cochlea; V = vestibule; dx = diagnosis; AIED = autoimmune inner ear disease; MD = Meniere’s Disease; SSNHL = sudden sensorineural hearing loss; HI-NOS = hearing instability not otherwise specified. ***p < 0.001, **p < 0.01, *p < 0.05.

Generalized linear mixed model (GLMM) analysis revealed significant associations between volume parameters and 4-frequency pure tone average (PTA). Variables were selected based on clinical relevance and correlation analysis (Supplemental Fig. 7). Given strong correlations between volume parameters, only one volume parameter, E/P ratio, was chosen for inclusion. Marginal and conditional R-squared values were both 0.46. Further details including Akaike Information Criterion (AIC), coefficients, standard error, t-value, and p-value for all fixed effects are reported in Supplemental Table 5. Higher vestibular E/P ratio positively influenced PTA (*p* < 0.001), indicating that greater endolymphatic volume in the vestibule is associated with worse hearing thresholds. SSNHL diagnosis showed a weaker effect that was not considered clinically meaningful due to a small cohort of patients with this diagnosis. MD also showed a marginal significant effect (*p* = 0.02). No other factors were significant. Scaled coefficients with 95% confidence intervals of all variables included in the model are shown in Fig. 3A.

These relationships were further examined by comparing E/P ratios when stratifying for improvement or worsening of hearing and correlating hearing to inner ear fluid volumes for stable instances and unstable instances separately. Figure 3B shows that E/P ratio was significantly higher in ears with worsening hearing compared to stable ears (Kruskal–Wallis *H* statistic = 9.51, *p* = 0.0086; adjusted Mann–Whitney *U* statistic = 131, *p* = 0.011). Compared to stable ears, visit-to-visit E/P ratio change was more negative in ears with improved hearing (Kruskal–Wallis *H* statistic = 12.41, *p* = 0.0020; adjusted Mann–Whitney *U* statistic = 787, *p* = 0.0082). Compared to ears with improved hearing, visit-to-visit E/P ratio change was more positive in ears with worsened hearing and (adjusted Mann–Whitney *U* statistic = 20, *p* = 0.026). When separating instances of stable hearing and unstable hearing, cochlear E/P ratios did not show a relationship with PTA (Supplemental Fig. 6A; Pearson’s correlation: *r*=-0.19, *p* = 0.07 for stable instances and Supplemental Fig. 6B; Pearson’s correlation: *r* = 0.025, *p* = 0.91 for unstable instances). However, vestibular E/P ratios showed significant positive correlation to PTA for unstable instances (Fig. 3C; Pearson’s correlation: *r* = 0.75, *p* < 0.001), which was stronger than the correlation for stable instances (Supplemental Fig. 8A; Pearson’s correlation: *r* = 0.41, *p* < 0.001). Low frequency (LF; 0.25, 0.5 kHz) or high frequency (HF; 4, 8 kHz) PTA showed similar correlations to E/P ratio as four frequency PTA (Supplemental Fig. 9; Pearson’s correlation: *r* = 0.77, 0.75 respectively, *p* < 0.001).

Vestibular E/P ratio was also compared to visit-to-visit cumulative threshold shift, defined as the difference in hearing thresholds from the prior audiogram, summed for changes > 10dB HL at each frequency. Most values for threshold shift at instances with stable hearing were 0, which led to a negligible slope between threshold shift and E/P ratio change on the estimated correlation line (Supplemental Fig. 8B; Pearson’s correlation: *r* = 0.09, *p* = 0.39). For instances of unstable hearing, a significant, positive relationship was found between vestibular E/P ratio change and threshold shift (Fig. 3D; Pearson’s correlation: *r* = 0.61, *p* = 0.003). This analysis indicates a direct relationship between vestibular fluid volume measurements and hearing that is temporally linked during instances of unstable hearing, although the relationship is not maintained when hearing is stable.

## Discussion

The longitudinal data collection in this study allowed novel consideration of how inner ear fluid volumes change over time in patients with HI. Initial analysis of volume measurements aligned with previous literature showing an increased proportion of endolymph within the inner ear of patients with Meniere’s disease compared to unaffected patients^28,29^. Our analysis extended this relationship to show increased endolymph proportion in ears with MRI-determined EH and at unstable hearing timepoints compared to HVs. Significantly higher variances for ears with fluctuating hearing loss and ears with MRI-determined EH indicate that fluid volumes are not only elevated, but also fluctuate in patients with HI over time (Fig. 2). Therefore, these volume measurements may provide additional clinical information beyond a binary indication of presence or absence EH.

Furthermore, vestibular volumes showed significant relationships to hearing level, suggesting their value as a potential biomarker. Several studies have previously found a correlation between hydrops severity and PTA^15,27^. This was redemonstrated in GLMM which accounted for additional patient factors that affect hearing (Fig. 3). Beyond unstable hearing being more prevalent when EH was observed on MRI, the extent of hearing deterioration was more pronounced with higher E/P ratios and shifts to better or worse hearing were associated with more negative or positive E/P ratio change respectively. Additionally, volume measurements have been shown to respond to treatment-related changes in Meniere’s disease in a study which found a decrease in endolymph volume following endolymphatic sac drainage and steroid therapy^30^.

Cochlear E/P ratios did not show strong differences or trends when comparing patient cohorts nor did they contribute significantly to relationships between volume and hearing measures (Figs. 2 and 3). Cochlear endolymph is difficult to detect given the complex rotational geometry of its structure, which distributes the shape of the endolymph space such that it reaches or falls below the 1mm^3^ resolution limit of 3T MRI. This often results in areas of discontinuous endolymph signal detection, as observed in this study and previous work employing similar endolymph detection methods^15,31^. There is no available strategy to account for the error that these signal disruptions introduce to quantification of cochlear endolymph. Although endolymphatic hydrops has been shown to be present in the cochlea, particularly in patients with fluctuating hearing loss, these changes may remain below the threshold of detection with current imaging techniques. Therefore, the absence of strong cochlear E/P trends in our results may reflect imaging limitations rather than true lack of pathology.

A custom software and analysis pipeline for semi-automated quantification of endolymph and perilymph volume from CED-FLAIR MRI images was developed for the assessment of volume change over time. Several previous studies have used similar imaging techniques combined with different processing methods to capture volumetric measurements, but none have considered their effectiveness for measuring repeated imaging over more than two timepoints^15,27–36^. Gürkov et al. and Homann et al. showed reliability of this technique, reporting ICC of test-retest reliability as 0.99 and intra-observer reliability as 0.99 respectively^15,27^. Only small deviations from zero in total volume distance from the mean were found in this study which indicates reliable volume detection from repeated images over time. Strong ICCs between users shows that the protocol used in this study is consistent and therefore appropriate for use in analyzing longitudinal change in volume parameters.

Several limitations of the study prompt future directions for this ongoing clinical protocol. Continued recruitment of healthy volunteers will allow expansion of an image database that can be used for defining normative endolymph and perilymph volume values and training deep learning-based models for more clinically-applicable fully automated image analysis. The current cohort contains small numbers of patients with SSNHL or AIED, limiting phenotyping and analysis of nuances between these patient groups. In previous literature, patients with idiopathic SSNHL demonstrate EH less frequently than in MD or acute low-tone SNHL^9,37,38^. Earlier distinction between presentation of sudden hearing loss and initial MD can better direct management. Expansion of SSNHL patients in our dataset will allow validation of these prior observations and exploration of volumetric analysis as a differentiating biomarker. Validation in larger cohorts and across other clinical centers is also necessary to confirm these findings and support broader clinical application of EH measurement.

While the current analysis focused on auditory function, future work may also explore the comparison of vestibular E/P ratio to additional audio-vestibular measurements and clinical manifestations. Relationships with vestibular evoked myogenic potentials, electrocochleography, vertigo symptoms, and quality of life measurements, for example, may further clarify the clinical utility of endolymphatic volume measurements in tracking disease activity or treatment response.

Overall, this study successfully demonstrated longitudinal fluctuation of inner ear fluid volumes in patients with hearing instability. Although challenges remain in measuring cochlear endolymph due to signal discontinuity, the findings reveal that vestibular E/P ratios are dynamic and correlate with hearing level changes. This suggests that vestibular E/P ratio is a promising biomarker for hearing instability and demonstrates its potential for clinical use in patient phenotyping or therapeutic selection.

## Materials and methods

### Subjects

Clinical data were collected prospectively from August 2021 to December 2023 for HI patients and August 2021 to June 2024 for HVs. Patients who met at least one of the two criteria for HI were included in the study: fluctuating hearing defined as threshold shift ≥ 20dB HL at one frequency or ≥ 15dB HL at 2 or more frequencies on serial audiometry or sudden hearing loss defined as threshold shift ≥ 30dB at 3 consecutive frequencies^3,39,40^. Patients 18–80 years old with normal middle ear function and no conductive hearing loss were included. Exclusion criteria included pregnancy, inability to undergo MRI with gadolinium contrast, or presence of active outer or middle ear disease or anomalies, chronic otitis media, bilateral profound sensorineural hearing loss, or history of a central nervous system disorder. No limitations were placed on therapeutic intervention during the study, medications taken by affected participants are shown in Supplemental Fig. 2.

Of the initially recruited 23 affected patients and 14 HVs, only data from affected patients who completed at least 3 study visits and HVs with 1–2 visits were included in the analysis. Data from 2 HVs were excluded due to motion during imaging, resulting in a final dataset of 14 affected patients and 12 HVs. Left and right ears for each patient were included, regardless of diagnosis laterality. Both the affected and HV cohorts contained male and female participants in equal proportion. In this analysis, data from one ear at a specified visit is referred to as an ‘instance’ (e.g., left ear of patient 3 at visit 5 is one ‘instance’).

### Study approval

All patients were enrolled in an observational study for deep phenotyping (https://clinicalstudies.info.nih.gov/protocoldetails.aspx?id=000141-DC). The protocol (NCT04806282) was approved by the Institutional Review Board of the National Institutes on Deafness and Other Communication Disorders, and all subjects gave informed consent. All methods were performed in accordance with relevant guidelines and regulations including the Declaration of Helsinki.

### Data collection timeline

All testing was performed at the NIH clinical center. Patients were evaluated intermittently over 15 months with 6–8 visits during periods of stable and fluctuating hearing (Supplemental Fig. 1). Healthy volunteers completed either one visit at baseline or two visits, with the second at 3 months. A comprehensive history and physical exam was performed by a neurotologist (M.H.) prior to audio-vestibular testing and CED-MRI imaging. Patients classified with SSNHL were determined to have idiopathic SSNHL following comprehensive evaluation and exclusion of known causes, including Meniere’s disease, AIED, vestibular schwannoma, Cogan’s syndrome, and other autoimmune conditions.

### Audio-vestibular testing

Hearing evaluation included pure tone thresholds for air and bone conduction from 0.25 to 8 kHz and tympanometry. Hearing loss was characterized using a 4-frequency (0.5, 1, 2, 4 kHz), low frequency (LF; 0.25, 0.5 kHz), and high frequency (HF; 4, 8 kHz) pure tone average (PTA). Clinically significant SNHL was defined as air conduction PTA > 25dB HL with an air-bone gap ≤ 10dB.

Hearing was designated unstable at one time-point if a threshold shift ≥ 20dB HL at one or more frequencies or ≥ 15dB HL at 2 or more consecutive frequencies occurred compared to the prior audiogram^39^. Unstable hearing was considered improved if cumulative threshold shift was negative and worsened if the shift was positive. Hearing stability was unknown at baseline visits, which were therefore excluded from analysis of hearing stability.

### MRI imaging of the internal auditory Canal (IAC)

**Fig. 4:**
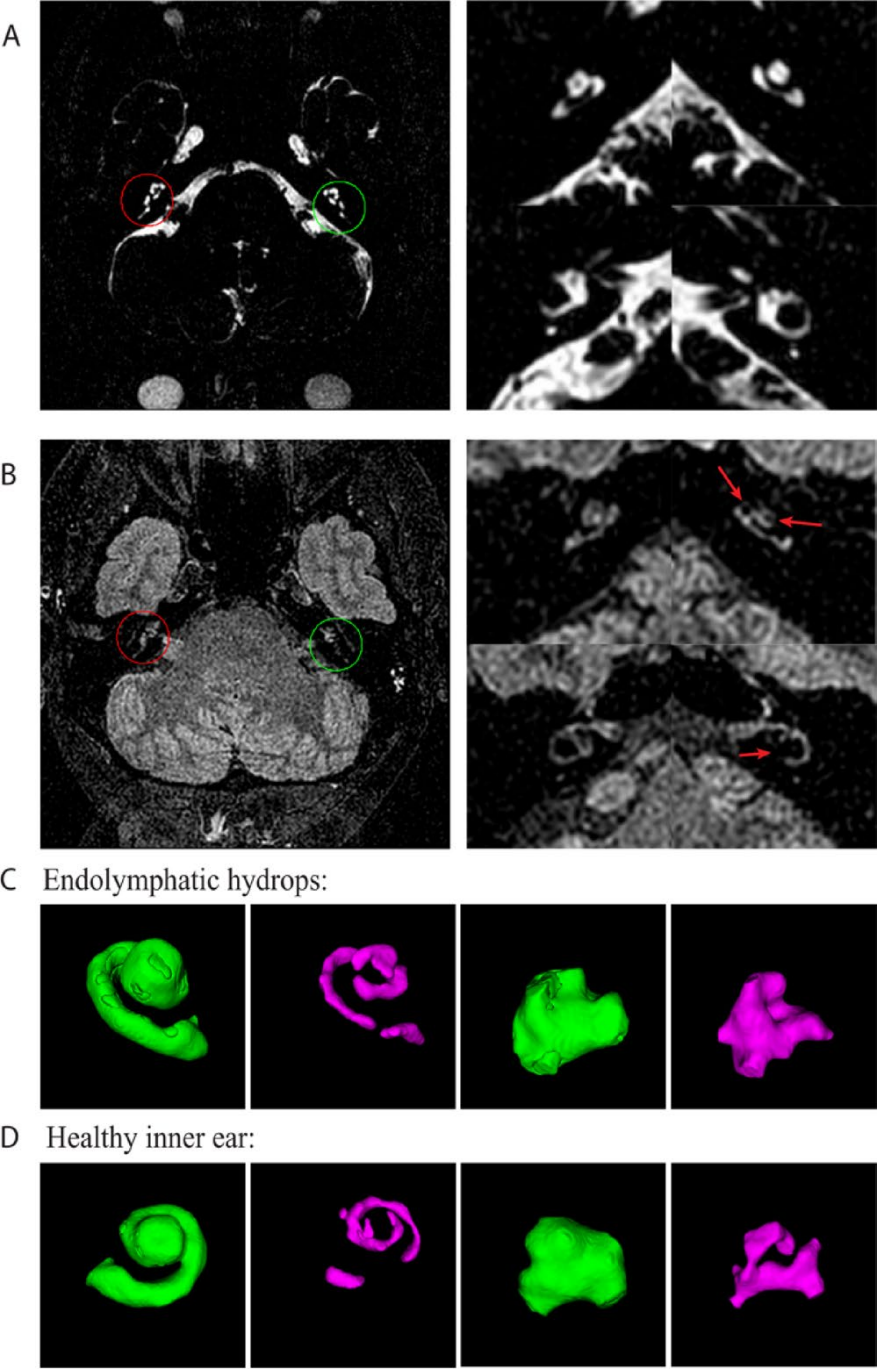
Example CED-FLAIR image from a patient with unilateral EH in the left cochlea and vestibule. (**A**) STIR images showing hyperintense total fluid. Image on the left shows an axial view of the cranium with inner ear structures circled in magenta (right ear) and green (left ear). On the right, enlarged images are shown of the cochlea in the top row and vestibule in the bottom row. (**B**) Corresponding FLAIR images to those in panel A in which perilymph only is hyperintense. Left FLAIR image shows axial view of the cranium with inner ear structures circled in magenta (right ear) and green (left ear). Enlarged images are shown on the right of the cochlea in the top row and vestibule in the bottom row. Presence of EH in the left ear is indicated by the enlarged dark areas within the cochlea and absence or thinning of perilymph signal behind the vestibule (red arrows). (**C**) Example images of hydropic inner ear 3D reconstructions are shown in panel C for the total volume in green and endolymph in magenta of the cochlea (left) and vestibule (right). (**D**) 3D reconstruction examples of inner ear structures from a healthy volunteer.

T2-weighted STIR and FLAIR MRI of the IAC were performed immediately prior to, immediately post, and 4–8 h after intravenous administration of gadoteridol (ProHance Bracco Diagnostics, Inc.; 0.2mmol/kg). Imaging was obtained using a 3.0T Philips Achieva MRI system with an 8-channel head coil at 0.5–0.8 mm isotropic resolution. All images were read by a neuroradiologist (J.B.) and evaluated for hyperintensity indicating increased protein on pre-contrast FLAIR images or inflammation on immediate post-contrast images. As perilymph appears hyperintense on CED-FLAIR, expanded hypointense endolymph space was used to designate endolymphatic hydrops on delayed images. Example imaging from a patient with left-sided EH (Fig. 4) compares total fluid seen on the STIR sequences (4A) to perilymph fluid in the FLAIR sequences (4B).

### Inner ear fluid volume quantification

A custom MRI processing and analysis software was developed to quantify total inner ear, perilymph, and endolymph volumes. The semi-automated pipeline and workflow is detailed in Supplemental Methods and Supplemental Fig. 3. The software was created using Interactive Data Language (IDL, NV5 Geospatial Solutions, Melbourne, FL, USA) and incorporated the DCM4CHE Java-based library for Digital Imaging and Communications in Medicine (DICOM) networking and file handling. 3D renderings of perilymph and endolymph are shown in Fig. 4C-D.

### Statistical analysis

Python (v3.11.0) was used for statistical analysis with the following libraries: NumPy (v1.24.3), Pandas (v1.5.3), Matplotlib (v3.7.1), Seaborn (v0.12.2), SciPy (v1.10.1), Statsmodels (v0.13.5)). Linear regression modeling was completed using R version 4.4.0 (2024-04-24; libraries used included lme4 (v1.1.35.3), lmerTest (v3.1.3), and car (v3.1.2)).

#### Volume quantification reliability

Reliability was assessed by evaluating TV deviations from individual ear volume means and by comparing analysis of TV and E/P ratio by two independent operators. Effect of gadolinium contrast dose and time from contrast administration to delayed MRI imaging (“delay time”) on perilymph volume detection was also examined (Supplemental Methods).

#### Characterization of longitudinal changes in fluid volumes

E/P ratios of the cochlea and the vestibule were used to analyze change in the endolymphatic compartment over time as this value encompasses both endolymph and perilymph fluid spaces and provides normalization for anatomical variation in inner ear size. Instances were grouped by hydrops status (EH or no EH) and by hearing stability (stable or unstable) and compared to HVs. Non-parametric testing with Kruskal-Wallis H test and post hoc-testing Mann-Whitney U test with Bonferroni correction was used to assess differences between groups to maintain consistency across all comparisons. To investigate how volumes fluctuated over time, E/P ratio variances across the study protocol were calculated for each ear, and median variance of ears with at least 1 EH visit or 1 unstable hearing visit were compared to ears with no EH visits or all stable visits with Mann-Whitney U test.

A Generalized Linear Mixed Model (GLMM) with a Gamma distribution and log link function was used to evaluate predictive ability of endolymph volume parameters for 4-frequency PTA. Variables included in the model were selected based on clinical relevance and analysis of correlation between variables. Random effects were included to account for variability of individual patients and left and right ears within patients. The model assumptions were verified, with no severe deviations in residuals, overdispersion, autocorrelation or multicollinearity. Random effects were normally distributed and sensitivity analysis confirmed model robustness.

## Electronic supplementary material

Below is the link to the electronic supplementary material.

Supplementary Material 1

Supplementary Material 2

## Data Availability

Deidentified individual participant data including demographic information, volume measurements, and hearing level will be indefinitely shared in supplemental eTable 1-3. Researchers may access these data without restrictions.
