# Supplementary material for: MRI-based measurement of inner ear fluids reveals increased endolymph volume variability in patients with endolymphatic hydrops and hearing instability
Source: Sci Rep. 2025 Jul 2;15:22560. doi: 10.1038/s41598-025-06083-w (PMC12217502; doi:10.1038/s41598-025-06083-w)
Supplement: Supplementary file 2 — Supplementary Material 2 [file 41598_2025_6083_MOESM2_ESM.docx]

**SUPPLEMENTAL MATERIALS**

**Contents**

1. Supplemental Methods
2. Supplemental Analysis
3. Supplemental Figures
4. Supplemental Tables
5. **Supplemental Methods**

*Semi-automatic Volume Quantification Pipeline*

Delayed post-contrast real STIR and real FLAIR images were pre-processed with manual selection of two circular regions of interest (ROI) containing the left and right inner ear structures. The ROIs were copied to all slices of the MRI which contained the cochlea, vestibule, or semicircular canals. A Contrast-Limited Adaptive Histogram Equalization (CLAHE) filter was then applied to the ROIs to improve image contrast. Corresponding STIR and FLAIR ROIs were then registered to align the structures of the two images.

Following image registration, segmentation was performed with automatic intensity thresholding on the delayed STIR image to identify total inner ear fluid. Pixel intensity distribution of the image was plotted, in which low intensity signal represented noise. A gaussian curve was fit to the low intensity signal distribution, the peak of which was used to select the lower limit of non-noise pixel intensities. The upper limit of included pixel intensity was set to 2.5% of the maximum pixel intensity. Threshold was then set at 50% of this intensity range to create a mask of the brightest structures in the image stack. The vestibulocochlear nerve also appears bright on STIR imaging, so this structure along with any extra erroneous structures was manually erased from the mask.

A difference image of the ROI was created by subtracting the STIR image (hyperintense total fluid) by the FLAIR image (hyperintense perilymph) to obtain an image with hyperintense endolymph signal. The total fluid mask was then mapped onto the difference image and a 50% intensity threshold was applied to pixels within the total fluid to obtain a mask of endolymph volume alone. 3D volume reconstructions were then automatically created from the stacks of area masks.

The 3D reconstruction of the total labyrinth was manually sectioned into specific regions of interest: cochlea and vestibule together, cochlea alone, and vestibule alone. Total volume and endolymph volume values were extracted from the 3D models.

*Reliability and effect of gadolinium contrast dose and imaging delay*

Repeatability was assessed using total volume (TV) measurements, and reliability was assessed using both TV and endolymph to perilymph (E/P) ratios. TV is expected to remain stable across visits for individual ears and can be used to test repeatability of manual sectioning of total labyrinth 3D reconstructions into the vestibule and cochlea. The difference between each visit’s TV value and the average value across visits for each individual ear was calculated (referred to as the distance from the mean) for both the cochlea and vestibule. To a evaluate reliability, two operators independently performed volume quantification including TV and E/P ratios for all visits of 4 selected patients. Resulting volume values were compared using a two-way random-effects intraclass correlation (ICC)*.*

Perilymph volume was used to assess effect of imaging protocol factors on volume detection. Instances were grouped by diagnosis status (affected or unaffected ear), presence or absence of EH, and hearing stability to visualize any relationship between contrast administration factors and volume measurements. In addition to dose and delay time alone, perilymph volumes were compared to a combined factor of dose multiplied by delay time (referred to as “dose-delay”).

1. **Supplemental Analysis**

***Supplemental Analysis 1: Significance of abnormalities on pre- and immediate post-contrast MRI imaging***

Neither hyperintense signal attributable to elevated fluid protein on pre-contrast FLAIR nor abnormal labyrinthine enhancement attributable to inflammation on immediate post-contrast FLAIR was associated with differences in hearing stability (Fisher’s Exact Test: odds ratio=0.51, 95% CI [0.11, 2.43], *p*=0.52; odds ratio = 0.56, 95% CI [0.12, 2.65], *p*=0.73) or EH presence on delayed imaging (Fisher’s Exact Test: odds ratio=1.26, 95% CI [0.46, 3.44], *p*=0.79; odds ratio=1.06, 95% CI [0.37, 3.04], *p*>0.99) compared to instances without abnormal early-imaging enhancement. Number of instances (N) for each comparison are in Supplemental Table 1. Findings on early sequences are therefore not informative of hearing presentation, whereas EH on delayed imaging indicated higher likelihood of unstable hearing.

***Supplemental Analysis 2: Inner ear fluid quantification reliability***

TV measurements, normalized as distance from the mean per individual ear, deviated by approximately 10 μL (Supplemental Figure 4A). Cochlear TV variance differed marginally between HV and affected data (Fligner-Killeen test: *p*=0.02) with no significant difference in variance for vestibular TV (Fligner-Killeen test: *p*=0.62). This low variance indicates that the analysis pipeline reliably detects inner ear structures in both healthy and affected ears.

TV and E/P ratios of four patients (8 ears) at each visit were compared between two independent operators (Supplemental Figure 4B). Values obtained by the two operators were well correlated with best-fit line slopes >0.8 for TV and E/P ratio in the vestibule and cochlea (Supplemental Figure 4B). Intraclass correlation coefficients from two-way random-effects models for E/P ratio and TV indicated good to excellent reliability (cochlea E/P ratio ICC=0.90, vestibule E/P ratio ICC=0.88; cochlea TV ICC=0.86, vestibule TV ICC=0.82). These correlations show that 3D volumes are sectioned consistently into discrete structures and the endolymph is detected reliably between operators.

1. **Supplemental Figures**

**Supplemental Figure 1: Schematic of study protocol.** Patients underwent an initial, 3-month, final (15 month) visit, and one additional visit per 3-month interval. Patients were instructed to notify the research team if they experienced a subjective change in their hearing and underwent testing within four days of the event. If no fluctuation event occurred, they were tested at the end of the 3-month time block (6, 9, or 12 months). If a fluctuation event occurred between the baseline and 3-month visit or the 12-month and final visit, patients were seen for an additional visit during those time blocks for a total of 6-8 per participant.


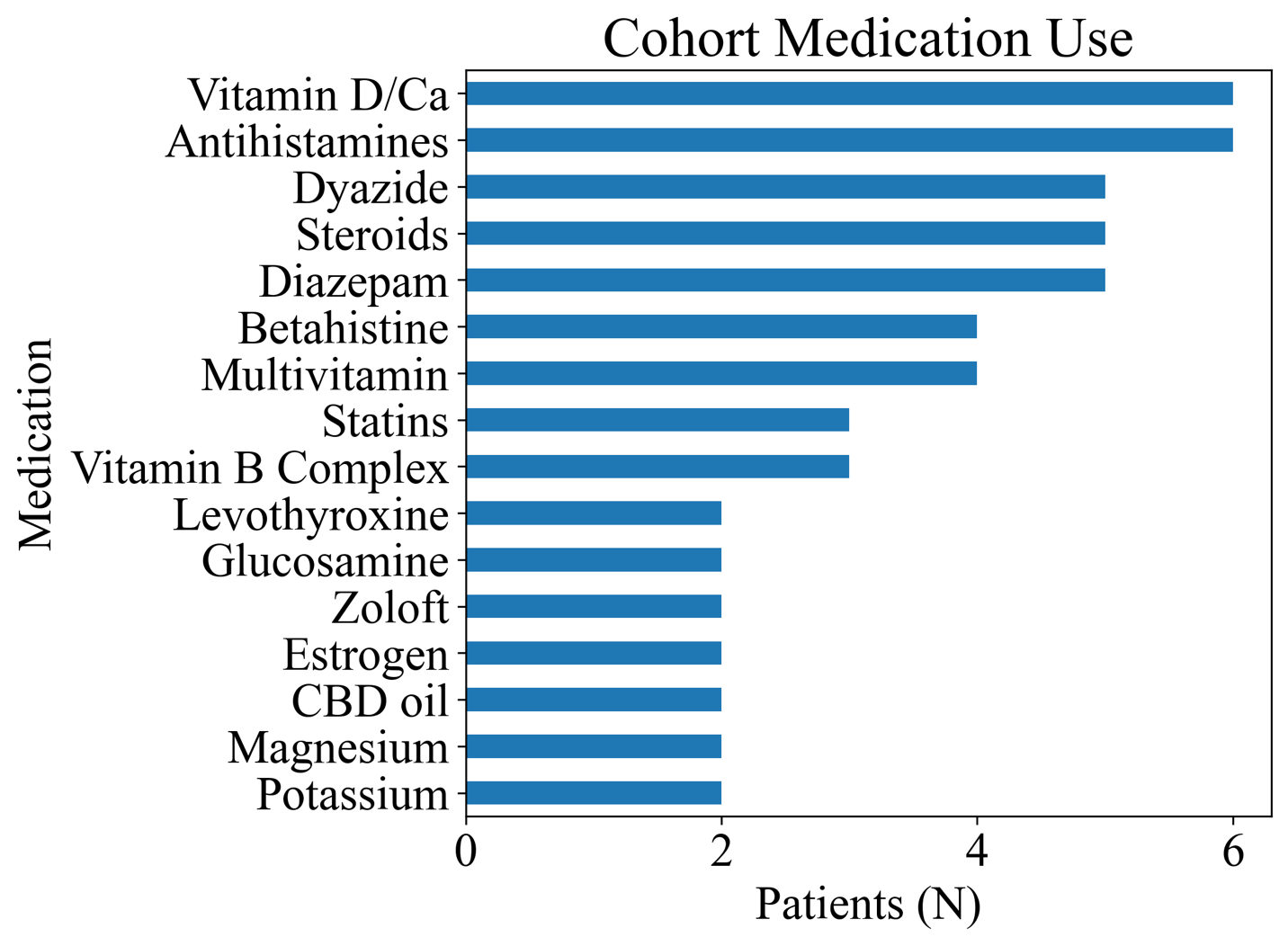


**Supplemental Figure 2: Summary of medications.** Medications taken by two or more patients in the cohort.


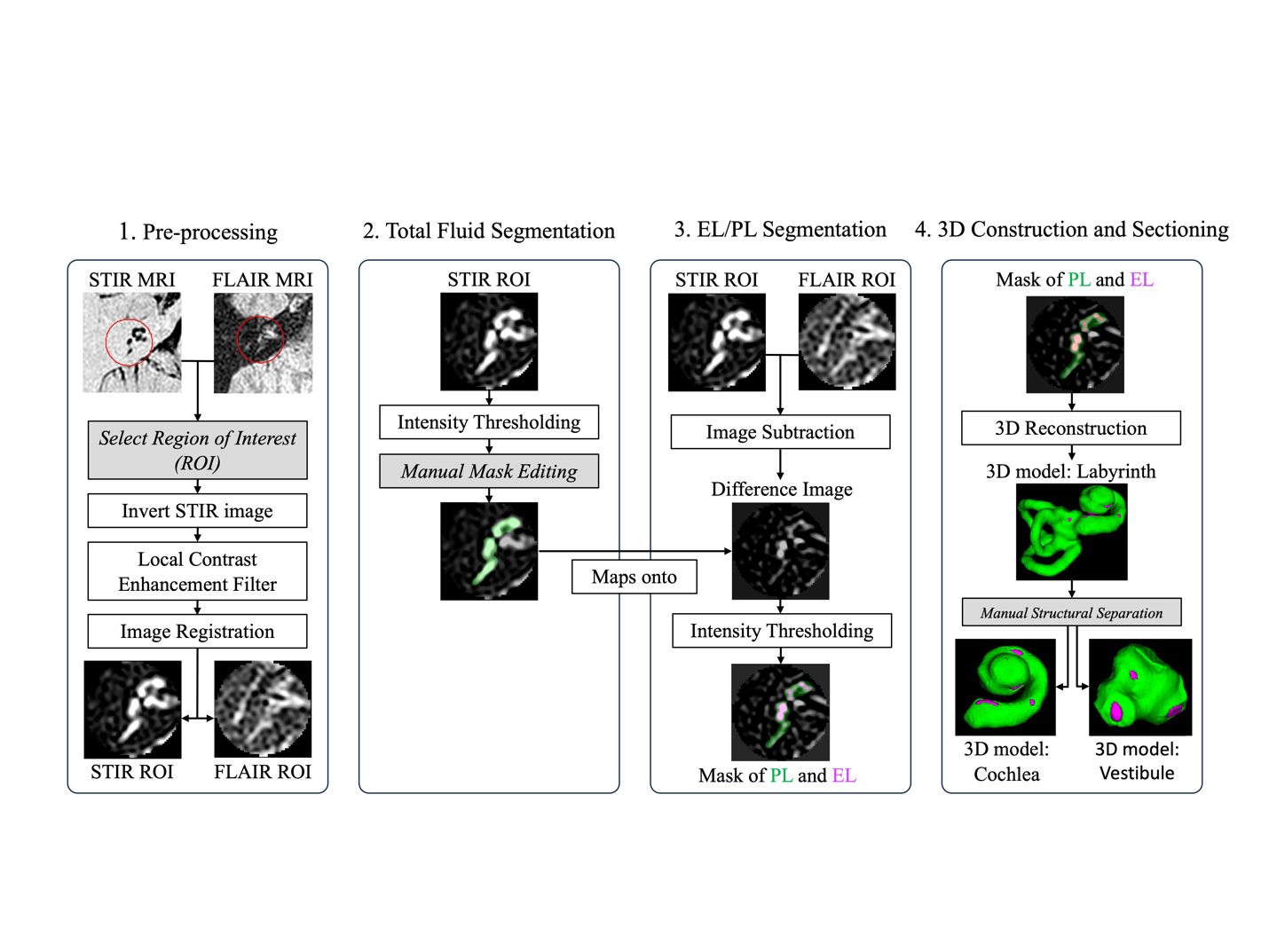


**Supplemental Figure 3:** **Volume quantification pipeline.** Visual representation of the four major steps in the volume quantification pipeline. Tasks that require manual input are highlighted in gray. EL = endolymph; PL = perilymph; ROI = region of interest


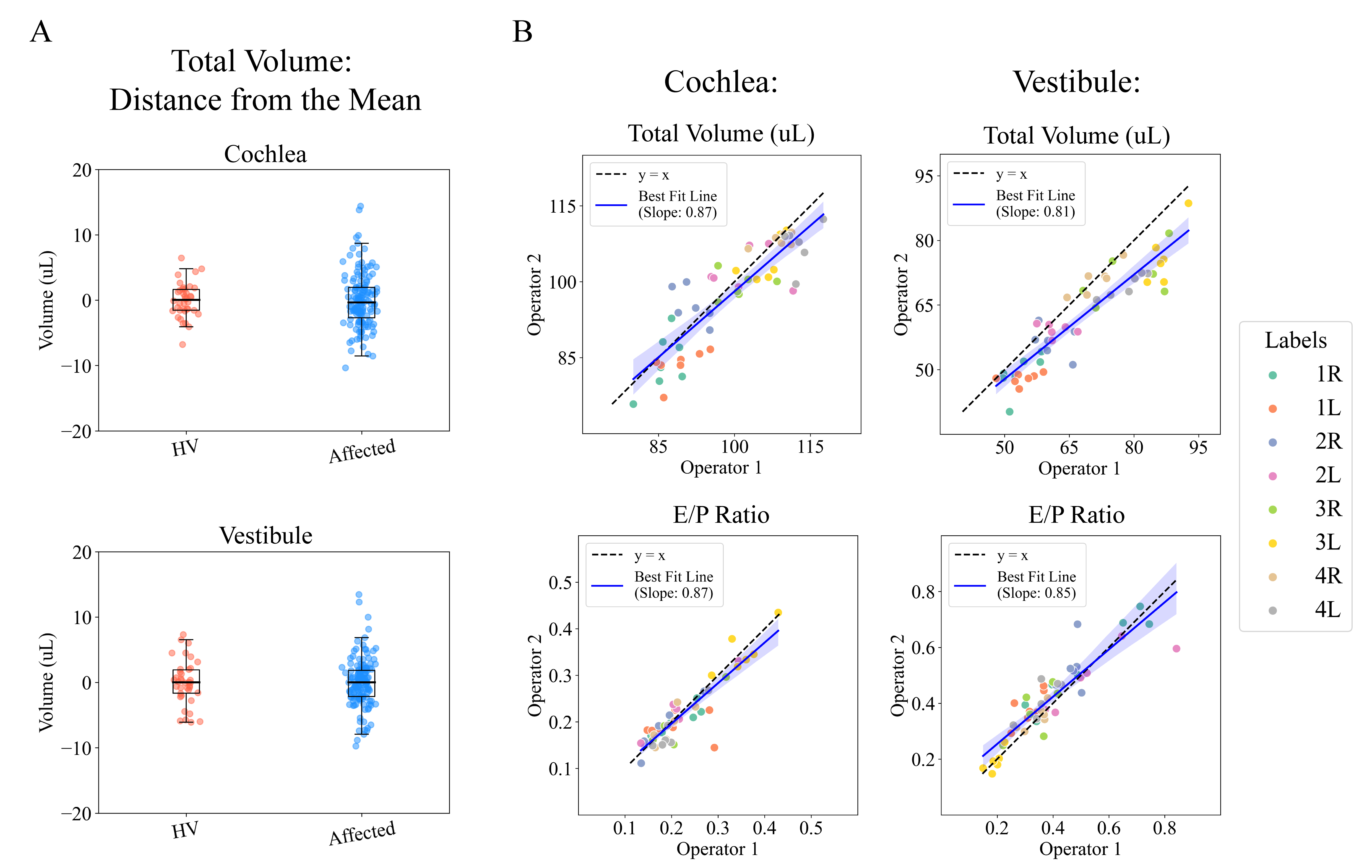


**Supplemental Figure 4:** **Image analysis pipeline reliability.** A) Comparison of total volume distance from the mean between HV and affected instances. Variance was marginally higher in affected vs HV cochlear data (HV variance=6.7, affected variance=17.2; Fligner-Killeen test: p=0.02), but not different between groups in the vestibule (HV variance=11.2, affected variance=13.2; Fligner-Killeen test: p=0.62). B) Correlation of total volume and E/P ratio measurements by operator 1 and operator 2 on the same MRI images for the cochlea (left) and vestibule (right). Datapoints are color labeled by individual ear as indicated by the legend on the right. Intraclass correlation coefficients from a two-way random-effects model for E/P ratio was 0.90 for the cochlea and 0.88 for the vestibule. For total volume, ICC was 0.86 for the cochlea and 0.82 for the vestibule.

**
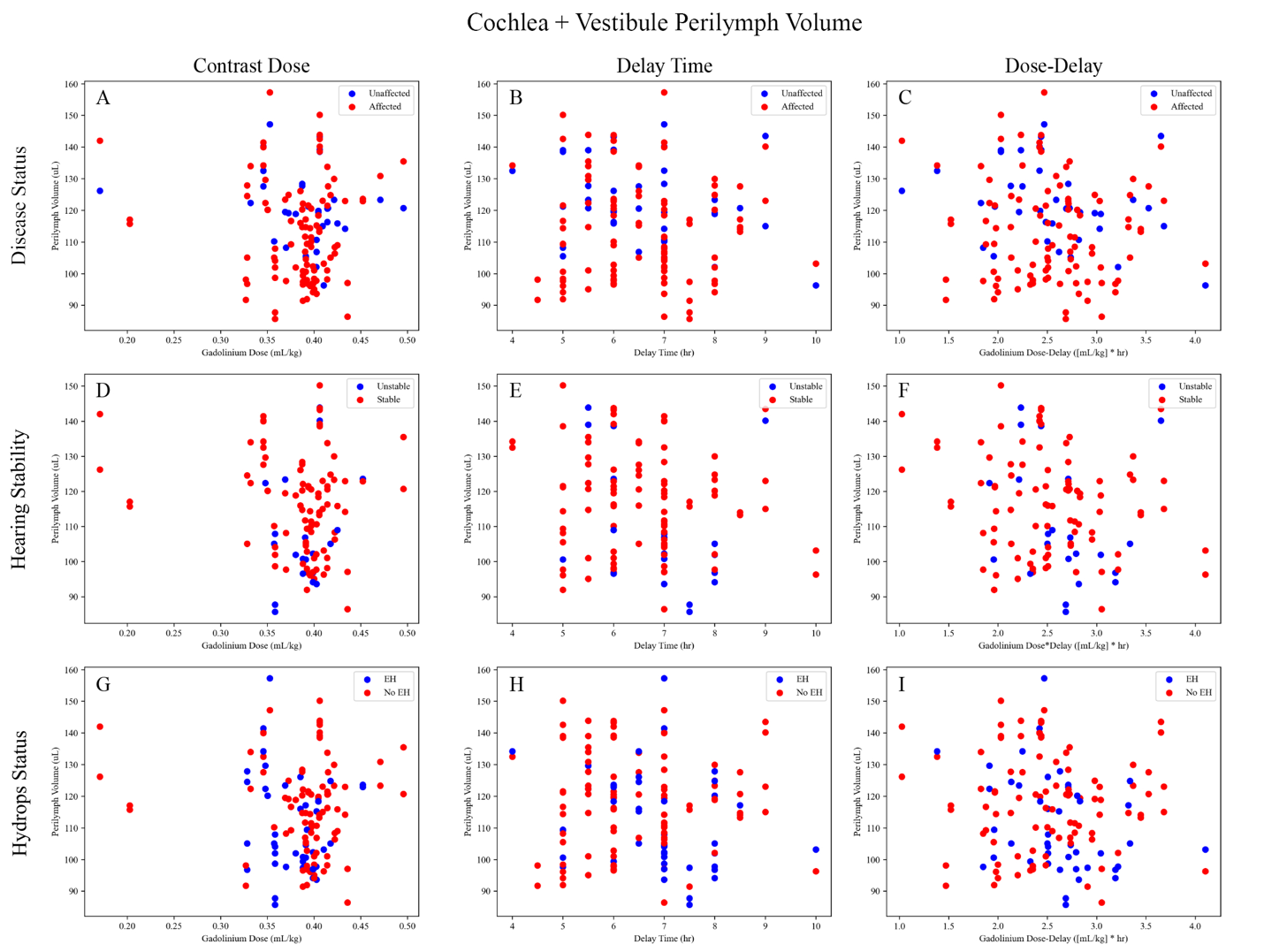
**

**Supplemental Figure 5:** **Impact of contrast administration on volume measurements.** Contrast dosing was adjusted for patient weight with a target of 0.4 mL/kg (0.2 mmol/kg). Patients received an average of 0.39±0.05 mL/kg of gadoteridol (range: 0.17-0.5 mL/kg). Delay time from contrast administration to MRI imaging ranged from 4-10 hours with an average of 6.59±1.22 hours. Comparisons of contrast administration factors including contrast dose (left column), delay from contrast administration to MRI imaging (delay time; middle column), and a combined parameter of dose multiplied by delay time (dose-delay; right column) to combined cochlear and vestibular perilymph volume for all instances are shown here. Scatter plots showing are labeled for ear disease status (A-C), hearing stability (D-F), and MRI-determined EH status (G-I). No relationship between perilymph volume and any of the three contrast administration factors is seen.

*
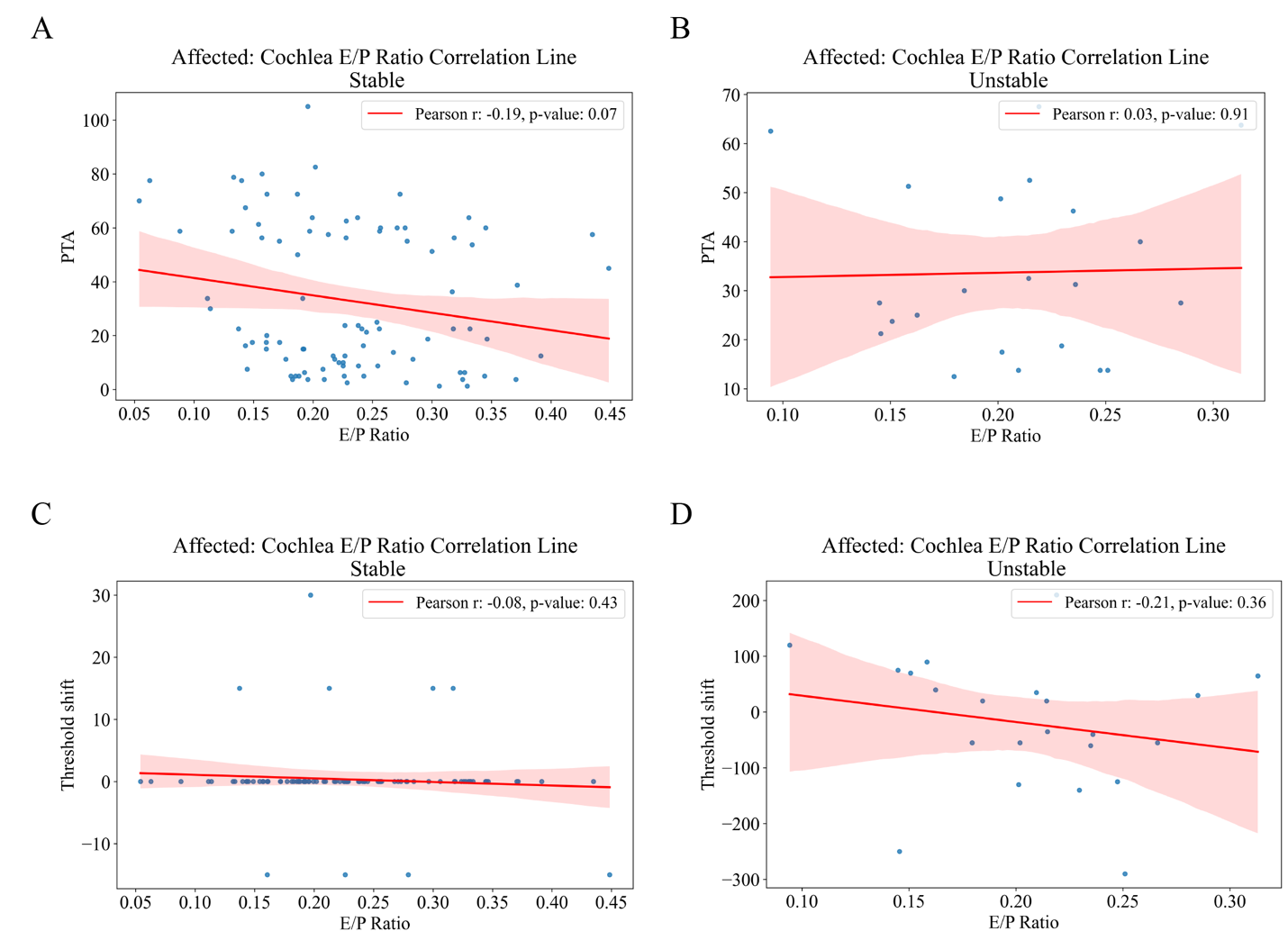
*

**Supplemental Figure 6: Correlations of cochlear volumes to hearing measures.** A-B) Correlation between cochlear E/P ratio and pure tone average (PTA) for instances with stable hearing (A; Pearson’s correlation: r=-0.19, p=0.068) and unstable hearing (B; Pearson’s correlation: r=0.025, p=0.91). C-D) Correlation between cochlear E/P ratio and threshold shift for ears with stable hearing (C; Pearson’s correlation; r= -0.08, p=0.43) and unstable hearing (D; Pearson’s correlation: r=-0.21, p=0.36). Visit-to-visit threshold shift was calculated as the sum of hearing threshold differences larger than 10dB HL for each frequency compared to the most recent prior audiogram.

**
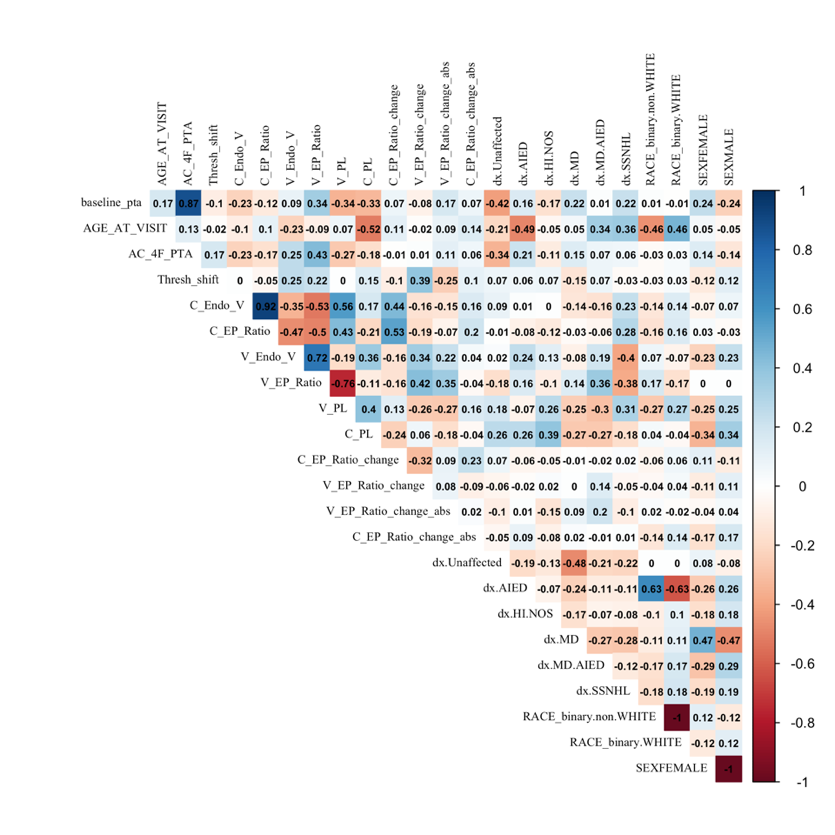
**

**Supplemental Figure 7:** **Correlation matrix.** Relationships are shown between all variables considered for inclusion in the regression models. Moderate to strong associations between several volume parameters as indicated by darker red and blue squares (r=0.92 for cochlear endolymph volume and E/P ratio; r=0.72 between vestibular endolymph volume and E/P ratio; r=-0.76 between vestibular E/P ratio and perilymph volume) lead to inclusion of only one volume parameter (E/P ratio for cochlea and vestibule) in the models. AC_4F_PTA=air conduction four frequency pure tone average; Thresh_shift=threshold shift; C_Endo_V=cochlea endolymph volume; C_EP_Ratio=cochlea endolymph/perilymph ratio; V_Endo_V=vestibule endolymph volume; V_EP_Ratio=vestibule endolymph/perilymph ratio; V_PL=vestibule perilymph volume; C_PL=cochlea perilymph volume; C_EP_Ratio_change=cochlea endolymph/perilymph ratio change; V_EP_Ratio_change=vestibule endolymph/perilymph ratio change; V_EP_Ratio_change_abs=vestibule endolymph/perilymph ratio change absolute value; C_EP_Ratio_change_abs=cochlea endolymph/perilymph ratio change absolute value; dx=diagnosis - AIED=autoimmune inner ear disease, HINOS=hearing instability not otherwise specified, MD=Meniere’s disease, SSNHL=sudden sensorineural hearing loss

**
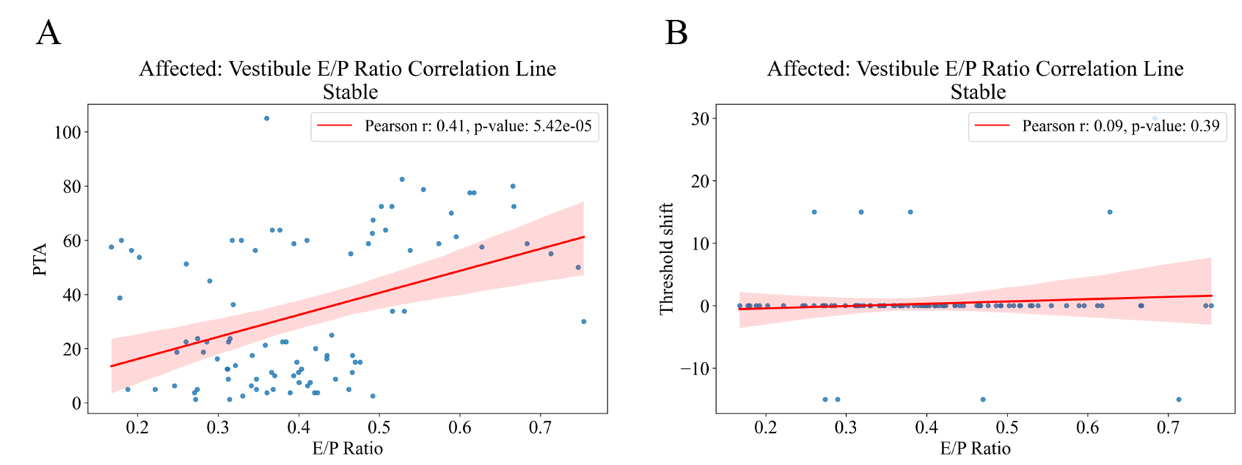
**

**Supplemental Figure 8: Correlation of E/P ratio to PTA for stable instances.** A) Correlation between vestibular E/P ratio and pure tone average (PTA) for instances with stable hearing (Pearson’s correlation: r=0.41, p=5.42e−05). B) Correlation between vestibular E/P ratio and cumulative threshold shift for ears with stable hearing (Pearson’s correlation; r=0.09, p=0.39).

*
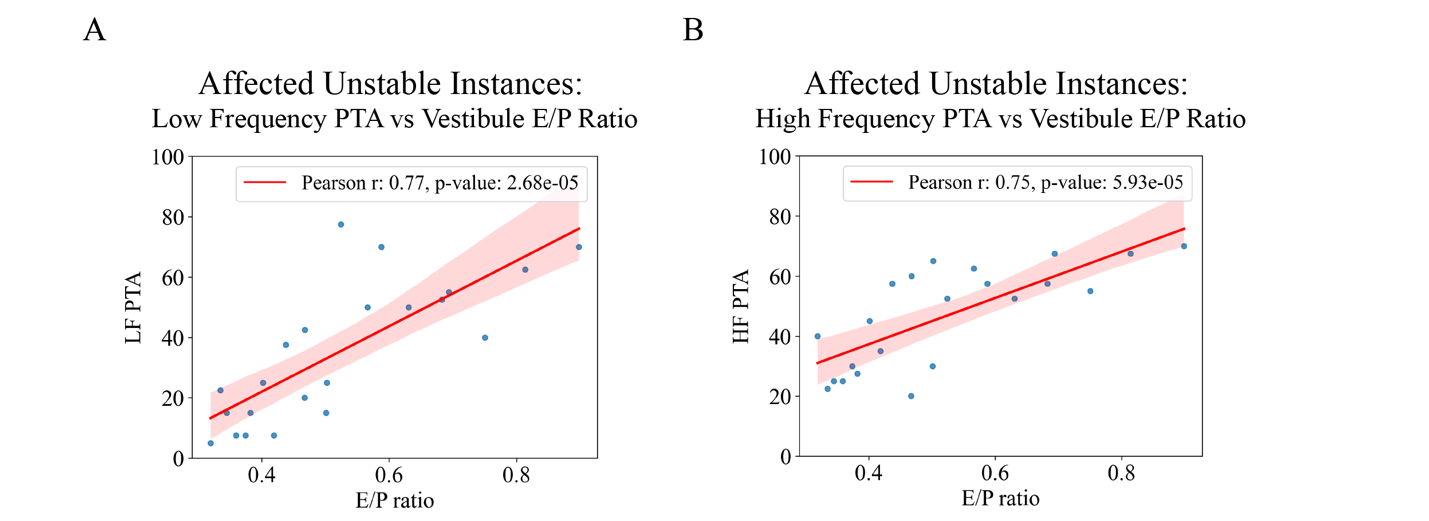
*

**Supplemental Figure 9: Correlation of E/P ratio to LF and HF PTA for unstable instances.** A) Correlation between vestibular E/P ratio and low frequency pure tone average (LF PTA) for instances with stable hearing (Pearson’s correlation: r=0.77, p<0.001). B) Correlation between vestibular E/P ratio and high frequency (HF) PTA for ears with unstable hearing (Pearson’s correlation; r=0.75, p<0.001). The correlation lines produced for LF and HF PTA are similar to that for four frequency PTA.

1. **Supplemental Tables**

**Supplemental Table 1: Correlations of components present on MRI imaging.** A) Number of stable vs unstable hearing instances with or without CED-FLAIR MRI-indicated EH, protein on pre-contrast FLAIR MRI, and enhancement (Enh) on immediate post-contrast FLAIR. B) Number of instances with vs without MRI-indicated EH with protein on pre-contrast and enhancement on immediate post-contrast imaging. Of note, one patient was unable to undergo immediate post-contrast imaging at their baseline visit. Therefore, two ears are not represented in the corresponding columns of sub-table B.

1. *Stability comparison*

|  | **Delayed Contrast Enhanced** | | **Pre-Contrast** | | **Immediate Post-Contrast** | |
| --- | --- | --- | --- | --- | --- | --- |
|  | EH | No EH | Protein | No Protein | Enh | No Enh |
| Unstable | 15 | 7 | 0 | 22 | 2 | 20 |
| Stable | 23 | 69 | 13 | 79 | 14 | 78 |

1. *EH comparison*

|  | **Pre-Contrast** | | **Immediate Post-Contrast** | |
| --- | --- | --- | --- | --- |
|  | Protein | No Protein | Enhancement | No Enhancement |
| EH | 7 | 39 | 6 | 39 |
| No EH | 12 | 84 | 12 | 83 |

**Supplemental Table 2: Summary of all volume measurements.** Overview of mean, standard deviation, and range of values for all inner ear fluid volume measurements obtained from CED-FLAIR MRI 3D volume reconstructions. Values from previous literature are included in the last two rows. EL = endolymph; E/T = endolymph to total volume; E/P = endolymph to perilymph; EH = endolymphatic hydrops noted on MRI.

|  | **# Instances** | **Cochlea** | | | | **Vestibule** | | | |
| --- | --- | --- | --- | --- | --- | --- | --- | --- | --- |
|  |  | **Total Volume (μL)** | **EL Volume (μL)** | **E/T Ratio** | **E/P Ratio** | **Total Volume (μL)** | **EL Volume (μL)** | **E/T Ratio** | **E/P Ratio** |
| no EH Mean | 96 | 91.91  ±12.46  (62.0-116.71) | 16.59  ±5.30  (2.95-29.74) | 0.18  ±0.05  (0.05-0.29) | 0.22  ±0.07  (0.05-0.41) | 58.24  ±9.56  (39.17-81.65) | 15.76  ±3.24  (8.09-23.30) | 0.27  ±0.05  (0.15-0.40) | 0.38  ±0.10  (0.18-0.67) |
| EH Mean | 46 | 90.40  ±10.55  (73.75-110.16) | 17.08  ±5.71  (6.9-33.38) | 0.19  ±0.05  (0.09-0.31) | 0.23  ±0.08  (0.09-0.45) | 55.61  ±8.64  (40.24-88.61) | 17.83  ±4.04  (10.8-27.03) | 0.33  ±0.09  (0.13-0.47) | 0.51  ±0.19  (0.15-0.90) |
| Stable Mean | 92 | 92.08  ±11.27  (66.96-116.71) | 17.18  ±5.75  (3.92-33.38) | 0.18  ±0.05  (0.05-0.31) | 0.23  ±0.08  (0.05-0.45) | 57.88  ±9.31  (39.17-78.32) | 16.07  ±3.48  (8.09-24.13) | 0.28  ±0.07  (0.14-0.43) | 0.41  ±0.13  (0.17-0.75) |
| Unstable Mean | 22 | 88.58  ±11.48  (73.75-107.64) | 14.96  ±3.27  (6.91-21.31) | 0.17  ±0.04  (0.09-0.24) | 0.21  ±0.05  (0.09-0.31) | 54.52  ±8.51  (43.11-71.19) | 17.99  ±2.93  (12.13-23.52) | 0.34  ±0.07  (0.24-0.47) | 0.52  ±0.16  (0.32-0.90) |
| Healthy Volunteer Mean | 38 | 93.18  ±10.47  (69.53-111.18) | 17.69  ±3.75  (10.85-25.02) | 0.19  ±0.032  (0.13-0.28) | 0.24  ±0.050  (0.15-0.39) | 59.57  ±8.67  (43.56-73.56) | 15.32  ±3.52  (8.71-23.18) | 0.26  ±0.047  (0.16-0.37) | 0.35  ±0.086  (0.18-0.59) |
| Previous reported average values: Healthy Patients | 16^31^  200^36^  126^41^  94^30^ | --  112.9±15.9  94.4±9.4  -- | 43.6 ±14.2  --  --  -- | 0.40±0.13  0.10±0.067  --  0.10±0.068 | --  --  --  -- | --  69.1±9.9  --  -- | --  --  --  -- | --  0.17±0.12  --  0.18±0.10 | --  --  --  -- |
| Previous reported average values: Non-Healthy Patients | 16 (MD)^31^  41 (LF-SSNHL)^30^  82 (sudden deafness)^30^  48 (MD no vertigo)^30^  72 (MD)^37^  22 (MD)^27^ | --  --  --  --  --  -- | 52.1 ±16.4  --  --  --  --  102 | 0.47±0.14  0.19±0.083  0.20±0.11  0.23±0.14  0.36±0.24  -- | --  --  --  --  --  -- | --  --  --  --  --  -- | --  --  --  --  --  -- | --  0.16±0.056  0.18±0.069  0.19±0.087  0.24±0.15  -- | --  --  --  --  --  -- |

**Supplemental Table 3: Statistical testing for E/P ratio comparisons by hydrops status. *adjusted with Bonferroni correction**

| **Volume Parameter** | **Value** | **Mean comparison** | | | | |
| --- | --- | --- | --- | --- | --- | --- |
|  |  | **Kruskal-Wallis H Test** | | **Post-Hoc Mann-Whitney U-Test** | | |
|  |  | **Statistic** | ***P*-Value** |  | **Statistic*** | ***P*-Value*** |
| Cochlea E/P Ratio | Raw Volume | 0.41 | 0.81 | - | - | - |
| Vestibule E/P Ratio | Raw Volume | 22.16 | 1.50E-05 | HV - No EH | 1038 | 0.79 |
|  |  |  |  | HV - EH | 433 | 2.60E-05 |
|  |  |  |  | No EH - EH | 769 | 9.84E-04 |

**Supplemental Table 4: Statistical testing for E/P ratio comparisons by hearing stability status. *adjusted with Bonferroni correction**

| **Volume Parameter** | **Value** | **Mean comparison** | | | | |
| --- | --- | --- | --- | --- | --- | --- |
|  |  | **Kruskal-Wallis H Test** | | **Post-Hoc Mann-Whitney U-Test** | | |
|  |  | **Statistic** | ***P*-Value** |  | **Statistic*** | ***P*-Value*** |
| Cochlea E/P Ratio | Raw Volume | 1.80 | 0.41 | - | - | - |
| Vestibule E/P Ratio | Raw Volume | 17.13 | 1.90E-04 | HV - Stable | 1018 | 0.18 |
|  |  |  |  | HV - Unstable | 143 | 4.30E-05 |
|  |  |  |  | Stable - Unstable | 399 | 0.025 |

**Supplemental Table 5: GLMM model predicting PTA.** Results of the GLMM evaluating the relationship between four frequency PTA and sex (ref=female), race (ref=non-white), diagnosis(ref=unaffected), age, cochlear E/P ratio, vestibular E/P ratio, and visit-to-visit cochlear and vestibular E/P ratio change (“E/P Ratio change”). Estimated coefficients, standard errors, degrees of freedom, t-values, and p-values for the fixed effects are shown. Variance component of random effects was 0.117±0.342 for individual patients and 0.118±0.343 for ears nested within each patient. Akaike information criterion (AIC) for this model was 1051.7, Bayesian Information Criterion (BIC) was 1093.1, Log-Likelihood was -511.9 and deviance was 13.2.

|  | **Estimate** | **Std. Error** | **t value** | **Pr(>\|z\|)** |
| --- | --- | --- | --- | --- |
| (Intercept) | 1.74315 | 1.358938 | 1.283 | 0.19959 |
| SEXMALE | -0.19008 | 0.564911 | -0.336 | 0.73651 |
| RACE_binaryWHITE | 0.410176 | 1.015138 | 0.404 | 0.68617 |
| dxAIED | 1.680684 | 1.419115 | 1.184 | 0.23629 |
| dxHI-NOS | 0.434997 | 0.816373 | 0.533 | 0.59414 |
| dxMD | 0.714013 | 0.309983 | 2.303 | **0.02126** |
| dxMD/AIED | 1.029502 | 1.095518 | 0.94 | 0.34735 |
| dxSSNHL | 1.487701 | 0.525433 | 2.831 | **0.00463** |
| AGE_AT_VISIT | -0.003895 | 0.022771 | -0.171 | 0.86418 |
| C_EP_Ratio | -0.065684 | 0.545962 | -0.12 | 0.90424 |
| V_EP_Ratio | 1.482075 | 0.285716 | 5.187 | **2.13E-07** |
